# Ilio-Colitis

**Published:** 1904-10

**Authors:** E. B. Parsons

**Affiliations:** Palestine, Texas


### TEXAS MEDICAL JOURNAL.

ESTABLISHED JULY, 1885.

PUBLISHED MONTHLY.—SUBSCRIPTION $1.00 A YEAR.

VOL. XX. AUSTIN, OCTOBER, 1904. No. 4.

### Original Contributions.

For Texas Medical Journal.

### Ilio-Colitis.—Etiology and Treatment.*

BY E. B. PARSONS, M. D., PALESTINE, TEXAS.

By ilio-colitis we mean an inflammation of the ilium and colon. But, as usually understood, it is a condition or morbid process which has continued from some gastro-enteric irritation until there have been produced marked lesion of the intestinal mucosa involving in part or all the walls of the intestines. Sometimes the transition from an acute attack of ptomaine poisoning, or from the exhibition of soprophytic bacteria contained in milk, it is almost impossible to draw the line between them, or where the ending of one or the beginning of the other. But, as this paper will deal with the sub-acute variety, we have as a pathological condition an intestine denuded of epithelium, a mucous and sub-mucous ulceration, congestion, and all the conditions found resulting from the inflammation of a mucous membrane.

In the light of modern research, I shall group all cases usually understood as catarrhal as "bacillary"; for, since 1889, Shiga of Japan, Krause of Germany, and Flexner of Philadelphia have found a bacillus identical in all cases of dysentery; and later, in 1901, Vedder and Duvall isolated the bacillus dysentericus in an alms house in New Haven, Conn. Other investigators have con-

*Read at meeting East Texas Medico-Chirurgical Society July, 1904.

firmed these reports, until we are forced to the conclusion that the bacillus dysentericus, if not the sole cause of ilio-colitis, or summer diarrhea, plays a very important rôle in its production. We can readily see what a suitable nidus we have in the intestine of a milk-fed baby, or in one in which indiscriminate eating, polluted water, foul air, infected milk, and a thousand different sources to depress the vitality of the young, decreasing the power of resistance of bacterial invasion, undigested food, producing irritation of the intestinal mucosa, is just the thing to invite a bacillus; and, once planted under the above condition, it multiplies rapidly, until the entire tract is a veritable culture tube. The limits of this paper forbid my going into the etiology of ilio-colitis. The various classifications, treatments, etc., may be best brought out by discussion; but, reasoning from the above causation of ilio-colitis, we would naturally conclude that, if the bacillus dysentericus is the cause of the disease, and of which I am confident, the remedy should be found in its specific antidysenteric serum, and I hope at no distant day to be able to treat all such cases with a serum. We are now possessed with one for practical use, and I hope in the future to report good results from its use. Much may be done by prophylaxis to limit the spread of this disease. First, by diet—and by diet I mean pure food, avoiding all extremes, clean bottles, sterilized milk, pure water, etc. If the disease is germ produced, it is capable of being transmitted by insects (flies). All babies suffering from the malady should be kept isolated from the flies. Dejecta should be disinfected immediately; houses screened; mosquito bars provided for all beds, as I firmly believe that house flies are responsible in a great measure for outbreaks of colitis in children. It is a fact beyond dispute that they carry the infection of typhoid fever, and it is reasonable to suppose they could carry the infection of ilio-colitis. We have been groping in the dark for ages, but, thanks to the men of modern bacteriological methods, light is being thrown on the difficult problems, and a brighter future is dawning. "'Tis a consummation devoutly to be wished" by the general practitioner, who watches anxiously over the lives of the little ones, the futures hopes of the country.

### TREATMENT.

In the treatment of this disease, the first consideration should be given to dietetics. Proteids and fats in the beginning should be eliminated, and in their place albumen, barley water, oat water, etc., should be substituted. Brandy freely given, and, as the symptoms become less acute, a gradual return to milk diet, largely diluted with barley water, say four or five times its volume to dilute the fats and casein, and, if agreeable, peptonize it to further digest the casein. Beef juice now becomes the favorite diet, with a liberal supply of albumen water, either acidulated or mixed with a little brandy; gradually return to a more liberal diet as the stools will indicate—"medicinal." Having a disease produced by a specific bacillus, why not use a specific serum? We have not advanced far enough in this special field of therapeutics yet to be specific, but early in the course of the disease I should unhesitatingly use the serum as prescribed now, but I have not much confidence in it later, as, in nearly all the cases, we have a mixed infection, in which other cocci play an important part too well known by the clinician.

### ANTISEPTICS.

Very little has been accomplished by internal antiseptics; for, in view of the great length of the intestinal tube, which, even under normal conditions, abounds in micro-organisms, it is impossible to sterilize its contents completely. But much may be done by the exhibition of such remedies as will at least partially inhibit bacterial growth; and, again, a remedy potent enough to kill the bacilli would do harm to the intestinal tissues. And, again, owing to the complex digestive juices, we are not sure but that our remedies are converted into an innocuous compound by the time they reach the diseased parts. It is my plan to treat these cases by the administration of small doses of hydrarg. chloridi mitis as a preliminary. This to be followed by a liberal dose of ol ricini, in which an opiate is added if thought necessary to control tormina and tenesmus. By this means, we asepticize as much as possible with the calomel, and clear the tract with oil, sweeping out all toxic material. This to be followed by a mild astringent, antiseptic, emollient ciccatrizant protective material, of which an emulsion of French bismuth in acacia is my favorite. Alternate these as occasion demands, using as little opium as possible. Bismuth, to do good, must be given in large doses. Two drams in twenty-four hours to a 2-year-old child. If another internal antiseptic is called for, we have a number to select from. The sulphocarbolate and salol have served me well, but of late I am accustomed to give acetozon, largely diluted with water, say from five to twenty grains to a liter of water, according to age, and have the little patient to sip this during the day ad libitum. This has proven an admirable antiseptic in all bowel troubles in my hands. The febrile reaction should receive prompt antipyretic, of which cool water, varied to suit the condition, is best; but the temperature will not be excessive if the intestinal tract is kept clean, thereby inhibiting autotoxemia and fever. Stimulants for the debility, good Cognac, diluted, without sugar, given freely; and, in addition, it has a good effect on the great splanchnic areas. Atrophine is of especial service late in course of the trouble, where we see the general peripheral paralysis indicated by blueness of the skin, and loose goose-skin flesh, and the boat-shaped abdomen. Colonic flushings in the sub-acute and chronic varieties is our sheet anchor. Wash out the colon every day with a gallon of normal saline solution, and gradually reduce these to twice per week. Later an astringent should be added, of which there are a great many to select from. Individually, I select tannin, as it inhibits bacterial growth, and also precipitates their toxins, and is the basis of all vegetable astringent. An acid should be given almost continuously to render the contents of the lower tube in an acid condition, also, inhibiting bacterial growth. I prefer either hydrochloric or sulphuric, properly diluted in dose suited to age of child. We should recollect at all times not to over medicate. They are slow to get well, and we may err on this side of safety to that of polypharmacy. Complications must be constantly watched; for oftentimes a slight cough and abrupt rise of temperature may mean a broncho-pneumonia, bronchitis, nephritis, etc. Keep child suitably clothed; flannel soft over abdomen, inunction of cod liver oil over abdomen, and, last, if convalescence is established, a suitable iron tonic, of which I prefer peptomanganate of iron and manganese. These are a few general principles on which to rely; for, as I have said before, the limits of this paper forbid my going into detail, as each individual has his personal preference for therapeutic remedies. I merely give you a broad outline, which may be enlarged upon accordingly. The parts in this paper I wish to impress upon you are that ilio-colitis is germ produced; and that bacillus dysentericus is one of the causes, and the medium of contagion may be flies; proper isolation of cases and protection by bars and screens to prevent ingress and egress of flies and insects.

BIBLOGRAPHY.

Hall's Diseases Infant Childhood American Text-Book Disease Children; Osler Practice Medicine; Journal Medical Association; Ky. Medical Journal.

